# Estrogen Alleviates Myocardial Ischemia–Reperfusion Injury by Inhibiting NLRP3 Inflammasome-Mediated Pyroptosis

**DOI:** 10.1155/crp/1850200

**Published:** 2025-10-09

**Authors:** Jing Cheng, Yutong Li, Shichun Shen, Jianlong Sheng, Banglong Xu, Xiaochen Wang, Cheng Cheng, Fei He

**Affiliations:** ^1^School of Nursing, Anhui University of Chinese Medicine, No. 350 of Longzihu Road, Hefei 230012, Anhui, China; ^2^Laboratory of Geriatric Nursing and Health, Anhui University of Chinese Medicine, No. 350 of Longzihu Road, Hefei 230012, Anhui, China; ^3^Department of Cardiology, Second Affiliated Hospital of Anhui Medical University, No. 678 of Furong Road, Hefei 230601, Anhui, China; ^4^Department of Cardiology, First Affiliated Hospital of University of Science and Technology of China, No. 1 of Tian'e Hu Road, Hefei 230036, Anhui, China; ^5^Department of Cardiology, Fist Affiliated Hospital of Anhui Medical University, No. 218 of Jixi Road, Hefei 230022, Anhui, China

**Keywords:** estrogen, myocardial ischemia/reperfusion injury, pyroptosis, signal pathway

## Abstract

**Background:**

The mechanism of estrogen-mediated myocardial protection remains incompletely understood. Our previous studies have shown that estrogen replacement therapy can modulate the expression of NLRP3 and alleviate inflammation in ovariectomized mice, while NLRP3 has been implicated in mediating cell pyroptosis. This study aimed to investigate the potential protective effects of 17β-estrogen (E2) on myocardial ischemia/reperfusion (I/R) injury by inhibiting NLRP3 inflammasome-mediated pyroptosis.

**Methods and Results:**

Using an ovariectomy (OVX) mouse model of myocardial I/R, our findings revealed that E2 replacement therapy led to a significant reduction in infarct size and pyroptosis levels, accompanied by a decrease in the expressions of key pyroptosis-related proteins including TXNIP, NLRP3, cleaved Caspase-1, ASC, IL-1β, and GSDMD. In vitro experiments with H/R cardiomyocytes further supported these observations, as E2 treatment improved cell viability and reduced pyroptosis-related protein levels. Conversely, coadministration of the estrogen receptor antagonist ICI 182780 reversed the protective effects of E2. Additionally, treatment with the NLRP3 inhibitor Bay11-7082 and the Caspase-1 inhibitor AC-YVAD-CMK also attenuated pyroptosis.

**Conclusions:**

Collectively, these results suggest that estrogen may alleviate myocardial I/R injury by inhibiting pyroptosis through the ER/TXNIP/NLRP3/Caspase-1 pathway, offering insights into potential therapeutic strategies for cardiac ischemic injury.

## 1. Introduction

Acute myocardial infarction (MI), commonly known as a heart attack, remains a major cause of death worldwide. It occurs when blood flow to a part of the heart muscle is blocked, usually due to a clot in the coronary arteries. This interruption in blood supply can lead to damage or death of the affected area of the heart. Reperfusion therapy, such as primary percutaneous intervention and thrombolysis, aims to restore blood flow to the heart after an acute MI. However, the process of resuming blood perfusion can paradoxically cause additional damage to the heart muscle, leading to myocardial ischemia/reperfusion (I/R) injury. This injury worsens the overall prognosis and increases the risk of complications following acute MI. Numerous experimental studies have identified various strategies for cardioprotection during ischemia and reperfusion, both through mechanical and pharmacological means. However, the translation of these findings into clinical practice has yielded disappointing results in many cases [1].Therefore, it is crucial to gain a deeper understanding of the mechanisms underlying myocardial I/R injury to develop effective strategies for preventing such damage and improving clinical outcomes.

Myocardial I/R injury entails a complex interplay of factors, including oxidative stress, inflammation, calcium overload, mitochondrial dysfunction, and the production of reactive oxygen species. Regardless of the specific mechanisms, programmed cell death (PCD) plays a significant role in the loss of cardiomyocytes during myocardial I/R injury. Among the different forms of PCD, apoptosis is widely recognized as a major contributor and is characterized by the activation of specific caspases, including Caspase-3. It is worth noting that pyroptosis, another form of PCD, has emerged as a distinct mechanism. Pyroptosis is triggered by certain microbial infections and cellular stressors, including I/R injury. Unlike apoptosis, pyroptosis distinguishes itself through unique molecular pathways and the release of proinflammatory cytokines [2, 3]. The hallmark of pyroptosis is its reliance on Caspase-1, which can be activated by a multiprotein complex known as the inflammasome. As a protease, activated Caspase-1 processes inactive precursors of interleukin-1β (IL-1β), interleukin-18(IL-18), and pro-Gasdermin D (GSDMD) into mature inflammatory cytokines. Notably, GSDMD possesses the ability to perforate the cell membrane, leading to cell lysis and subsequent death [4]. The NLRP3 inflammasome is presently the extensively studied inflammasome, comprising NLRP3 (nucleotide-binding domain, leucine-rich-containing family, pyrin domain-containing-3), ASC (apoptosis-associated speck-like protein), and Caspase-1 [5, 6]. Upon detection by NLRP3, damage signals can stimulate Caspase-1 cleavage and the release of proinflammatory cytokines [7]. A prior investigation showcased a significant elevation of NLRP3 following MI, which was correlated with an impaired cardiac function in mice exposed to I/R injury [8, 9]. Thioredoxin-interacting protein (TXNIP) is a member of the α-arrestin protein superfamily and engages in direct interaction with NLRP3, thereby playing a pivotal role in the activation of the NLRP3 inflammasome [10]. Deletion of TXNIP has been shown to confer protection against I/R injury [11]. Furthermore, previous study has uncovered that TXNIP-mediated NLRP3 inflammasome activation represents a novel mechanism underlying I/R injury in cardiac microvascular endothelial cells [12]. Emerging evidence confirms that TXNIP/NLRP3 signaling also contributes to cardiomyocyte injury during I/R [13, 14]. Therefore, targeting the inhibition of NLRP3 inflammasome activation via the blockade of the TXNIP/NLRP3 signaling pathway could be a promising therapeutic strategy for alleviating I/R injury.

It is widely acknowledged that women exhibit a lower incidence of MI and experience better prognoses compared to men at similar ages. However, this favorable trend undergoes a reversal following menopause. The underlying mechanisms contributing to the gender disparity in myocardial ischemia injury remain poorly understood. Studies have indicated that an early bilateral oophorectomy in females is associated with increased cardiovascular mortality, suggesting a potential cardioprotective effect of female sex hormones [15]. A wealth of evidence suggests that estrogen replacement therapy has the potential to alleviate myocardial I/R injury in female animals subjected to ovariectomy (OVX) [16–18]. Recent studies have demonstrated that estrogen could inhibit the activation of the NLRP3 inflammasome and reduce the production of proinflammatory cytokines. This suggests that estrogen may exert its cardioprotective effects, at least in part, through the modulation of NLRP3 inflammasome-mediated inflammation [19, 20]. Similarly, our previous research has revealed that 17 beta-estradiol (E2) significantly ameliorated airway inflammation by inhibiting the NLRP3 inflammasome and its downstream products, including Caspase-1 and the proinflammatory cytokine IL-1β [21]. Furthermore, reports indicate that E2 can downregulate the expression level of TXNIP by binding with ER-β, shedding light on a potential mechanism through which estrogen modulates the activity of TXNIP [22]. However, it remains unclear whether E2 could alleviate myocardial I/R injury through the TXNIP/NLRP3-mediated pyroptotic pathway. The present study was designed to explore the effects of E2 on TXNIP/NLRP3-mediated pyroptosis in the context of myocardial I/R injury, aiming to provide further insights into the potential mechanisms underlying the cardioprotective effects of estrogen. The results suggest that estrogen could potentially reduce myocardial I/R injury by suppressing pyroptosis via the ER/TXNIP/NLRP3/Caspase-1 pathway, providing valuable insights for developing therapeutic approaches for treating cardiac ischemic injury.

## 2. Materials and Methods

### 2.1. Animals and Groups

C57 female mice were purchased from the Animal Experimental Center of Anhui Medical University. All experimental operations were performed in compliance with the National Institutes of Health Guidelines for Care and Use of Laboratory Animals (Eighth Edition. Washington, DC: The National Academies Press. https://doi.org/10.17226/12910) and approved by the Ethics Committee of Anhui Medical University (Grant No. LLSC20200498).

**Figure 1:**
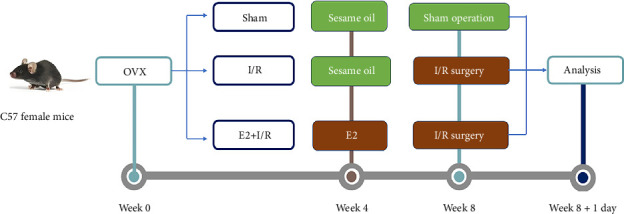
Experimental timeline of the in vivo animal study. C57BL/6 female mice underwent bilateral ovariectomy (OVX) at Week 0, followed by a 4 week recovery period. From Week 4 to Week 8, animals received daily subcutaneous injections of either 17β-estradiol (E2; 5 μg/mL in sesame oil) or vehicle control (sesame oil). At Week 8, myocardial ischemia/reperfusion (I/R) surgery was performed via transient ligation of the left anterior descending coronary artery (30 min ischemia/24 h reperfusion), with sham-operated controls undergoing identical procedures without ligation. Terminal analysis occurred at Week 8 + 1 day, encompassing infarct size quantification (TTC/Evans blue staining), serum biomarker assessment (LDH, CK-MB), and pyroptosis evaluation (TUNEL, Western blot for TXNIP/NLRP3 pathway proteins). Experimental groups included OVX + Sham (vehicle), OVX + I/R (vehicle), and OVX + E2+I/R (*n* = 14/group).

Experimental timeline and group allocation are shown in Figure 1. Mice were randomly assigned to the following three groups, and there are 14 mice in each group:

1. OVX + Sham heart surgery (Sham)
  The protocol of OVX has been described in detail previously [21]. Briefly, fasted mice were anesthetized with sodium pentobarbital (80 mg/kg) and underwent bilateral OVX via abdominal incisions. For heart surgery, a similar procedure was followed, including anesthesia, intubation, thoracotomy, and stringing, but without ligation of the anterior descending branch of the left coronary artery (LAD), serving as a control for assessing the effects of LAD ligation on myocardial I/R injury.
2. OVX + I/R; 4 weeks after successful OVX, mice in this group underwent coronary artery ligation to establish a myocardial I/R model, following a previously described method [23]. In brief, mice were adequately anesthetized with sodium pentobarbital and ventilated with a ventilator (HX-101E, Techman soft Co., Ltd., Chengdu, China) during the procedure. Thoracotomy was performed in the fourth intercostal space to expose the heart, and the LAD was transiently ligated for 30 min using a nylon monofilament (Lingqiao, Ningbo, China). Successful occlusion was confirmed by visual changes in the left ventricular wall and ECG alterations. Reperfusion was initiated by untying the ligation, as confirmed by the recovery of the ST-segment on the ECG.
3. OVX + E2 + I/R
  Prior to LAD ligation, mice in one group received subcutaneous E2 supplementation (5 μg/mL in sesame oil) for 4 weeks post bilateral OVX. The above two groups were given an equivalent volume of sesame oil without E2 subcutaneously.

### 2.2. Serum Levels of E2, LDH, and CK-MB

After reperfusion, blood samples were collected for the serum E2 level measurement using the E2 kit from Meilian, Shanghai, China. Additionally, LDH and CK-MB concentrations in the serum were analyzed using a biochemical analyzer from Siemens, Germany.

### 2.3. Comparison of MI Size

To determine the MI area, a 2% Evans blue solution (Sigma, US) was intravenously injected via the right internal jugular vein following reocclusion of the LAD. The heart was then rapidly frozen at −80°C for 10 min and sliced into 2 mm sections. These slices were incubated with 2% triphenyl tetrazolium chloride (TTC) in darkness at 37°C for 15 min. The area at risk (AAR), where viable cardiomyocytes were stained brick-red by TTC, while the infarcted myocardium remained white, was identified as the unstained portion of the myocardium with Evans Blue. The infarct size (IS) was calculated as a percentage of IS/AAR. Both IS and AAR were quantified digitally using Image J software (version 1.46r, National Institutes of Health, USA) and expressed as a percentage of the left ventricular area (IS/LV and AAR/LV).

### 2.4. Primary Cardiomyocyte Culture

Neonatal ventricular myocytes (NVMs) were isolated from the whole heart of 1–3 day-old C57 mice [24]. In short, heart was incubated with 0.25% trypsin (0.5 mL/sample) containing ethylene diamine tetraacetic acid (EDTA) overnight at 4°C for preliminary digestion. The next day, Dulbecco's modified Eagle's media (DMEM) supplied with 10% fetal bovine serum (FBS) was applied to neutralize trypsin. Then, supernatant was discarded and added with 0.1% type II collagenase for further digestion (0.6 mL/heart) at 37°C several times until no visible tissue. After centrifugation, NVMs were resuspended in DMEM containing 10% FBS and antibiotics. Differential adhesion method was applied to remove myocardial fibroblasts. In the first 24 h, bromodeoxyuridine (Brdu, 1 mM, sigma) was added into the medium to inhibit fibroblasts [23].

### 2.5. In Vitro Experiment Groups

NVMs were randomly allocated to the following groups:

1. Control group
  NVMs were cultured constantly under normal condition.
2. Hypoxia–reoxygenation (H/R) group
  NVMs were incubated with mixed gas containing 95%N_2_ and 5%CO_2_ at 37°C for 6 h and then moved to an incubator with normal conditions for 12 h.
3. H/R + E2(10 nM); NVMs in this group were administrated with 17β-estrogen (E2, 10 nM) 24 h before H/R procedure.
4. H/R + E2+ ICI 182780 (ICI) (100 nM); to explore the underlying mechanism by which E2 confers cardioprotective effects, NVMs in this group were coadministered with estrogen receptor antagonist ICI 182780 (ICI) at a nontoxic concentration (100 nM) 24 h before the H/R procedure.
5. HR + E2 +ICI + Bay11-7082 (5 uM)
  To demonstrate that E2 inhibits pyroptosis via NLRP3 inflammasome, NVMs in this group were treated with a specific NLRP3 inflammasome inhibitor Bay11-7082 at 5 uM for 24 h before H/R.
6. HR + E2+ ICI + AC-YVAD-CMK (50 μM)
  To demonstrate that E2 could inhibit pyroptosis, NVMs in this group were treated with Caspase-1 inhibitor AC-YVAD-CMK at 50 uM for 24 h before H/R.

### 2.6. Cell Viability Assay

Cell viability was measured with CCK-8 kit (Beyotime) according to the manufacturer's instruction. NVMs were cultured in 96-well plates at a density of 5 × 10^4^ cells per well. Then, NVMs were cultured with 10 μL CCK8 for 2 h. Absorbance was measured at 450 nm using a microplate reader [Molecular Devices (MD), US)]. Cell survival rate was calculated using the mean optical density (OD) of each group. The formula was: cell survival rate = OD of the treatment group/OD of the control group × 100%.

### 2.7. The Determination of Lactate Dehydrogenase (LDH) Activity

LDH activity in the medium was measured using a colorimetric assay kit (Nanjing Jiancheng, China) according to the manufacturer's instructions.

### 2.8. The Determination of Caspase-1 Activity

Caspase-1 activity is detected by a commercial kit (Solarbio, Beijing, China). The detection kit is based on the Caspase-1 catalytic substrate acetyl-tyrosine alanine p-nitroaniline (AC-YVAD-pNA) to generate yellow p-nitroaniline (pNA), with strong absorption at 405 nm. Thus, the activity of Caspase-1 can be assessed by measuring the absorbance of pNA using a standard pNA curve.

### 2.9. Il-1, IL-18, and TXNIP Tests

Levels of IL-1, IL-18, and TXNIP released in a cultured supernatant were determined by enzyme-linked immunosorbent assay (ELISA). IL-1, IL-18, and TXNIP levels were measured according to the manufacturer's instructions in the IL-1 (Meilian, Shanghai, China), IL-18 (Meilian, Shanghai, China), and TXNIP (Meilian, Shanghai, China), respectively.

### 2.10. TUNEL (Cardiomyocyte Immunofluorescence)

After the myocardial I/R procedure, mouse hearts were removed, paraffin-embedded, and sectioned at the ligation site. TUNEL assay was used to detect cell death that was measured in each group after the corresponding treatment [25]. The TUNEL assay kit (Roche, Germany) was used for detection, and the process followed the manufacturer's instructions. At the same time, DAPI (Beyotime, China) is a blue fluorescent dye that can strongly bind to the DNA in the cardiomyocytes, so we detected the content of cellular DNA based on its fluorescence intensity. These images were taken using a Nikon Eclipse Ti microscope (Nikon, Japan). The fluorescence intensity was recorded using a fluorescence microscopy (Olympus BX53, Japan). The cell death rate was calculated as (number of TUNEL positive cells/total number counted) × 100%.

### 2.11. Western Blot Analysis

Western blot analysis was performed as described previously using antibodies against TXNIP (Ab188865, Abcam), NLRP3 (1:1000, Abcam, Ab210491), apoptosis-associated speck-like protein containing a CARD (ASC) (1:200, Santa Cruz, sc-22514-R), Caspase-1 (1:200, Santa Cruz, sc-514), IL-1β (1:1000, Abcam, ab9722), Gasdermin D protein (GSDMD) (1:1000, Abcam, ab219800), and GAPDH (1:1000, CST, D16H11). GAPDH was used as a loading control [26]. The protein bands were detected with an Odyssey color infrared laser scan-imaging instrument (Li-Cor, USA).

### 2.12. Statistical Analysis

All values are expressed as mean ± standard deviation. One-way ANOVA analysis of variance was used to analyze the differences among each group, and then Bonferroni post-test, *p* < 0.05, indicates statistical significance; ImageJ version 1.53 (National Institutes of Health), GraphPad Prism version 6.0 (GraphPad Software, La Jolla, CA, USA), and Adobe Illustrator CC 2019 (Adobe Software, San Jose, CA, USA) were used to drawing.

## 3. Results

### 3.1. E2 Replacement Therapy Alleviates MI/R Injury In Vivo

**Figure 2:**
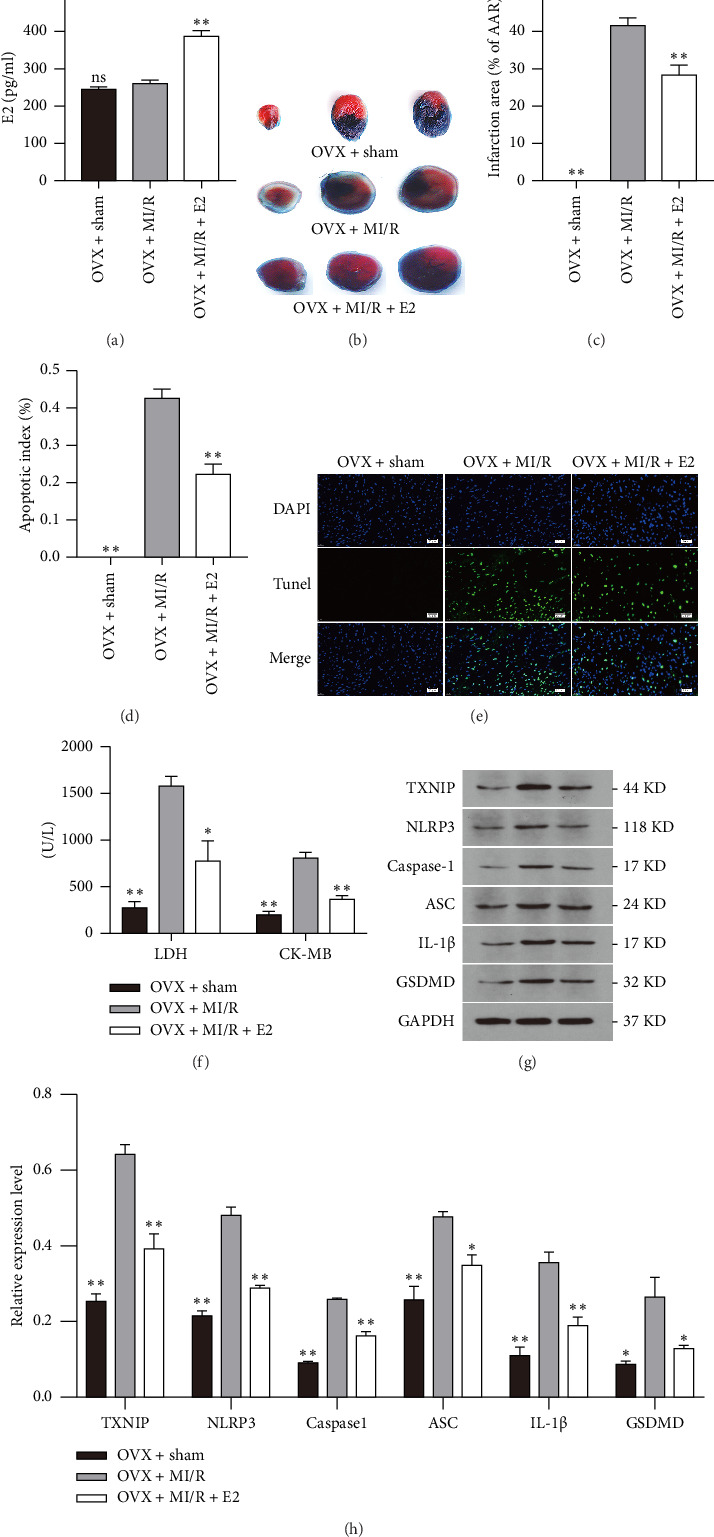
Comparison of plasma E2 level, infarct size, pyroptosis and pyroptosis-related proteins in mice (*n* = 10 in each group). (a) Plasma E2 level alteration induced by OVX and estrogen replacement. No significant difference of plasma E2 was observed after OVX between Sham and MI/R groups. E2 dramatically increased as a result of estrogen replacement therapy for 4 weeks. The results shown are mean ± SD from three independent experiments. ^∗∗^*p* < 0.01 compared with OVX + MI/R group; (b) representative TTC–Evans Blue stained sections of hearts from OVX + Sham, OVX + MI/R, and OVX + MI/R + E2 group. The area stained with Evans blue represented the non-I/R myocardium, whereas the unstained area was the I/R myocardium or AAR. Within the AAR, the brick red-stained area represented viable myocardium, whereas the unstained (white) area represented infarcted myocardium; (c) the ratio of IS/AAR. The results shown are mean ± SD from three independent experiments. ^∗∗^*p* < 0.01 compared with OVX + MI/R group; (d) comparison of TUNEL-positive cells in each group. TUNEL-positive cells significantly increased following MI/R. E2 replacement therapy significantly reduced TUNEL-positive cells. The results shown are mean ± SD from three independent experiments. ^∗∗^*p* < 0.01 compared with OVX + MI/R group; (e) representative micrographs of left mid-ventricular sections with TUNEL-staining (green), and total nuclei was stained by DAPI (blue). (f) Comparison of LDH and CK-MB in each group. Both enzymes dramatically increased following MI/R. E2 replacement therapy significantly reduced both enzymes. The results shown are mean ± SD from three independent experiments. ^∗^*p* < 0.05 compared with the OVX + MI/R group; ^∗∗^*p* < 0.01 compared with the OVX + MI/R group; (g) Expression of pyroptosis-related proteins in each group determined by Western blot. (h) Comparison of pyroptosis-related proteins in each group. These proteins dramatically increased following MI/R. E2 replacement therapy significantly reduced these proteins. The results shown are mean ± SD from three independent experiments. ^∗^*p* < 0.05 compared with OVX + MI/R group; ^∗∗^*p* < 0.01 compared with OVX + MI/R group.

In our previous study, we observed a significant reduction in plasma E2 to approximately 250 pg/mL after OVX, which then increased dramatically to 400 pg/mL as the result of estrogen replacement therapy. The present study further confirms a similar alteration in plasma E2 levels induced by OVX and estrogen replacement (Figure 2(a)).

IS was assessed using TTC/Evans blue staining, as depicted in Figure 2(b). Similar to previous studies, we observed that OVX mice exhibited a larger infarction size compared to sham-operated OVX mice (Figure 2(c)). Additionally, the ratio of TUNEL-positive cells in the OVX + I/R group was notably higher than in the sham-operated OVX mice (Figures 2(d) and 2(e)). Furthermore, plasma LDH and CK-MB levels were higher in the I/R group compared to those in the sham group (Figure 2(f)). Moreover, we evaluated the effect of estrogen on myocardial I/R injury and found that E2 replacement therapy significantly reduced IS, TUNEL-positive cells, as well as plasma LDH and CK-MB levels compared with mice in the OVX + I/R group (Figures 2(b), 2(c), 2(d), 2(e), 2(f)). The above experimental results indicate that OVX can lead to an increase in the MI size and cell death during I/R injury. However, E2 replacement therapy can reduce IS and decrease cell death.

### 3.2. E2 Replacement Therapy Inhibits NLRP3-Mediated Pyroptosis During Myocardial I/R Injury In Vivo

In our previous study, we have shown that E2 replacement therapy can downregulate the expressions of NLRP3, ASC, and Caspase-1. It is widely acknowledged that pyroptotic cell death can be initiated by the activation of Caspase-1 through various inflammasomes, including the NLRP3 inflammasome. In the present study, we assessed the protein expressions of genes related to pyroptosis. We observed significant increases in the levels of these proteins, including TXNIP, NLRP3, cleaved Caspase-1, ASC, IL-1β, and the pyroptosis executor GSDMD in the OVX + I/R group. However, E2 replacement therapy markedly suppressed the expressions of these proteins (Figures 2(g), 2(h)). The results suggested that E2 replacement therapy might alleviate myocardial I/R injury partly through inhibiting NLRP3-mediated pyroptosis.

### 3.3. E2 Treatment Alleviated H/R Injury via Estrogen Receptor Pathway In Vitro

**Figure 3:**
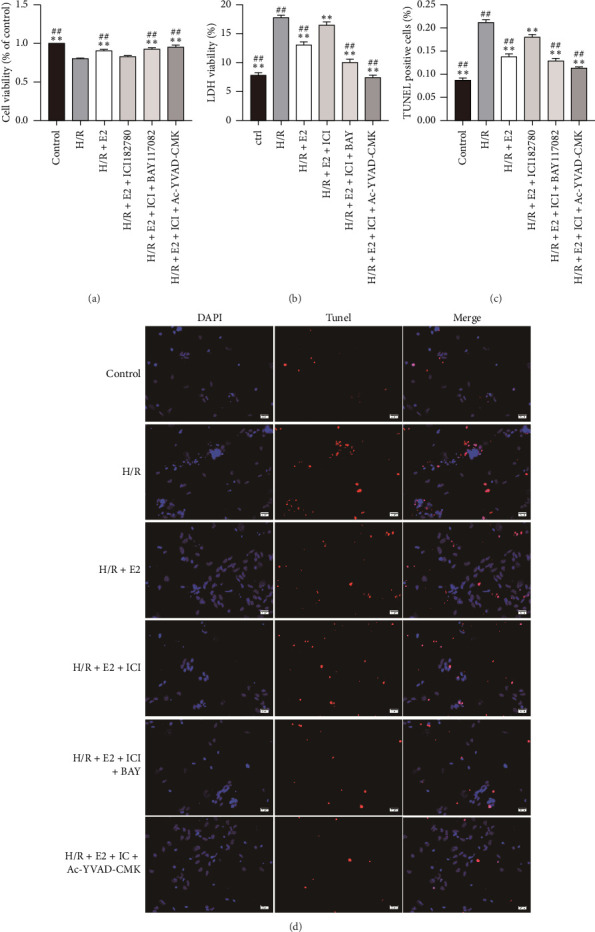
Comparison of cell injury TUNEL-positive cells in each group in primary cardiomyocytes (*n* = 6 in each group). (a) Comparison of cell viability in each group. Cell viability decreased significantly after H/R procedure. E2 pretreatment significantly preserved cell viability, which could be partly reversed by ER antagonist ICI 182780. Compared with the H/R + E2 + ICI 182780 group, pretreatment with either Bay11-7082 or AC-YVAD-CMK could significantly preserve cell viability. The results shown are mean ± SD from three independent experiments. ^∗∗^*p* < 0.01 compared with the H/R group; ^##^*p* < 0.01 compared with the H/R + E2+ICI 182780 group. (b) Comparison of LDH in each group. LDH increased significantly after H/R procedure. E2 pretreatment significantly reduced LDH, which could be partly reversed by ER antagonist ICI 182780. Compared with the H/R + E2+ICI 182780 group, pretreatment with either Bay11-7082 or AC-YVAD-CMK could significantly reduce LDH. The results shown are mean ± SD from three independent experiments. ^∗∗^*p* < 0.01 compared with H/R group; ^##^*p* < 0.01 compared with the H/R + E2+ICI 182780 group. (c) Comparison of TUNEL-positive cells in each group. TUNEL-positive cells increased significantly after H/R procedure. E2 pretreatment significantly reduced TUNEL-positive cells, which could be partly reversed by ER antagonist ICI 182780. Compared with the H/R + E2 + ICI 182780 group, pretreatment with either Bay11-7082 or AC-YVAD-CMK could significantly reduce TUNEL-positive cells. The results shown are mean ± SD from three independent experiments. ^∗∗^*p* < 0.01 compared with the H/R group; ^##^*p* < 0.01 compared with the H/R + E2 + ICI 182780 group. (d) Representative micrographs of NVMs with TUNEL-staining (red) and total nuclei were stained by DAPI (blue).

To investigate whether E2 exerts its cardioprotective effects through receptor or nonreceptor pathways, we performed in vitro studies utilizing primary cardiomyocytes subjected to H/R procedures to simulate I/R injury. Compared to cardiomyocytes in the control group, cell viability significantly decreased after 4 h of hypoxia followed by 2 h of reoxygenation (Figure 3(a)). Both the release of LDH and the TUNEL-positive cells increased dramatically compared to the control group, indicating significant cell death induced by the H/R procedure (Figures 3(b), 3(c), 3(d)). Consistent with the in vivo study, E2 pretreatment significantly preserved cell viability and decreased LDH release and TUNEL-positive cells in vitro. However, the protective effects of E2 administration on H/R injury were markedly abolished when simultaneously pretreated with the estrogen receptor antagonist ICI 182780, suggesting that E2 alleviates H/R-induced cell injury via the estrogen receptor pathway (Figures 3(a), 3(b), 3(c), 3(d)).

### 3.4. E2 Treatment Alleviated Pyroptosis via Estrogen Receptor Pathway In Vitro

**Figure 4:**
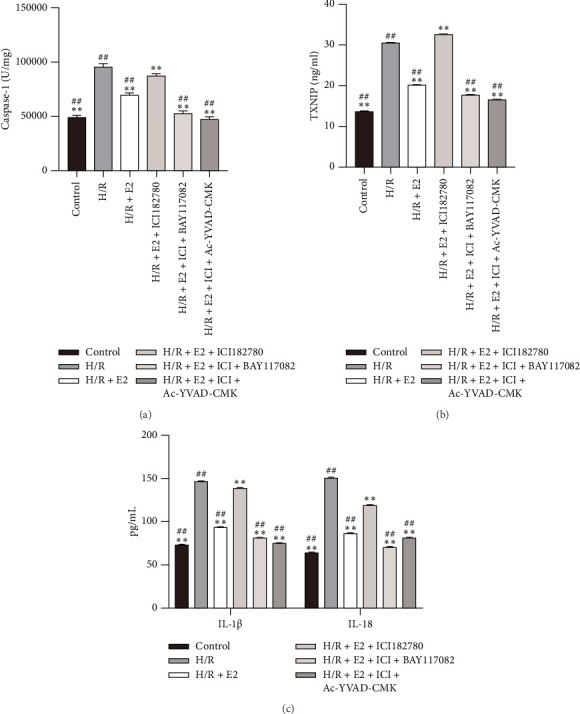
Comparison of Caspase-1 activity, TXNIP, IL-1β, and IL-18 in each group in primary cardiomyocytes (*n* = 6 in each group). (a) Comparison of Caspase-1 activity in each group. Caspase-1 activity increased significantly after H/R procedure. E2 pretreatment significantly reduces Caspase-1 activity, which could be partly reversed by ER antagonist ICI 182780. Compared with the H/R + E2+ICI 182780 group, pretreatment with either Bay11-7082 or AC-YVAD-CMK could significantly reduce Caspase-1 activity. The results shown are mean ± SD from three independent experiments. ^∗∗^*p* < 0.01 compared with the H/R group; ^##^*p* < 0.01 compared with the H/R + E2+ICI 182780 group. (b) Comparison of TXNIP in each group. TXNIP level increased significantly after H/R procedure. E2 pretreatment significantly reduces the TXNIP level, which could be partly reversed by ER antagonist ICI 182780. Compared with the H/R + E2 + ICI 182780 group, pretreatment with either Bay11-7082 or AC-YVAD-CMK could significantly reduce the TXNIP level. The results shown are mean ± SD from three independent experiments. ^∗∗^*p* < 0.01 compared with the H/R group; ^##^*p* < 0.01 compared with the H/R + E2 + ICI 182780 group. (c) Comparison of IL-1β and IL-18 in each group. IL-1β and IL-18 levels increased significantly after H/R procedure. E2 pretreatment significantly reduces IL-1β and IL-18 levels, which could be partly reversed by ER antagonist ICI 182780. Compared with the H/R + E2 + ICI 182780 group, pretreatment with either Bay11-7082 or AC-YVAD-CMK could significantly reduce IL-1β and IL-18 levels. The results shown are mean ± SD from three independent experiments. ^∗∗^*p* < 0.01 compared with the H/R group; ^##^*p* < 0.01 compared with the H/R + E2 + ICI 182780 group.

**Figure 5:**
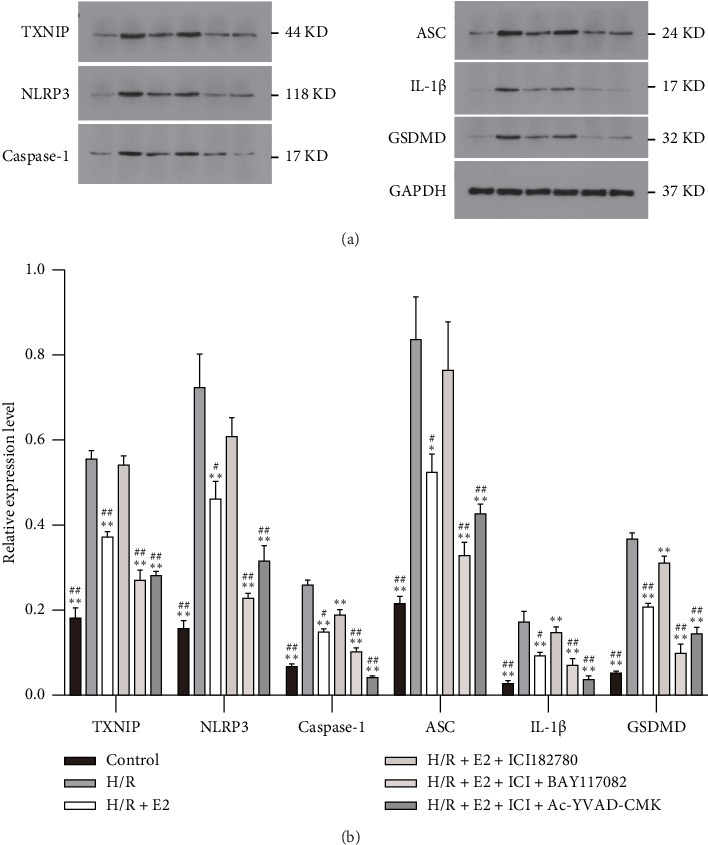
Comparison of pyroptosis-related proteins in each group in primary cardiomyocytes (*n* = 6 in each group). (a) Representative blots were displayed. (b) Comparison of pyroptosis-related proteins in each group. These proteins increased significantly after the H/R procedure. E2 pretreatment significantly reduces the expression of these proteins, which could be partly reversed by ER antagonist ICI 182780. Compared with the H/R + E2 + ICI 182780 group, pretreatment with either Bay11-7082 or AC-YVAD-CMK could significantly reduce the expression of these proteins. The results shown are mean ± SD from three independent experiments. ^∗∗^*p* < 0.01 compared with the H/R group; ^##^*p* < 0.01 compared with the H/R + E2 + ICI 182780 group.

To determine whether pyroptosis is involved in the cardioprotective effects of E2, we measured and compared the expression levels of pyroptosis-related genes in subsequent in vitro experiments. Consistent with the in vivo study, the activity of Caspase-1 significantly increased after 4 h of hypoxia followed by 2 h of reoxygenation, indicating an activation of Caspase-1 during the H/R procedure (Figure 4(a)). The release of TXNIP, IL-1β, and IL-18 also increased dramatically compared to that of the control group (Figures 4(a), 4(b), 4(c)). In addition, the expressions of pyroptosis-related genes including TXNIP, NLRP3, ASC, cleaved-Caspase-1, GSDMD, and IL-1β were noticeably elevated in the H/R group (Figures 5(a) and 5(b)). As expected, E2 pretreatment effectively reduced the activity of Caspase-1, as well as the release of TXNIP, IL-1β, and IL-18 (Figures 4(a), 4(b), 4(c)) as well as expressions of TXNIP, NLRP3, ASC, cleaved-Caspase-1, GSDMD, and IL-1β (Figures 5(a) and 5(b)). However, these protective effects were significantly reversed when coadministered with ICI 182780, indicating the involvement of estrogen receptors in the mediation of E2's protective effects against pyroptosis induced by H/R (Figures 4(a), 4(b), 4(c)). These findings suggest that the H/R procedure induces substantial pyroptosis, and E2 treatment may alleviate such pyroptosis via an estrogen receptor-dependent pathway.

### 3.5. E2 Treatment Therapy Alleviated H/R Injury Partly Through Suppressing Pyroptosis In Vitro

To further demonstrate the involvement of pyroptosis inhibition in the cardioprotective effect of E2, primary cardiomyocytes were pretreated with either the NLRP3 inflammasome inhibitor Bay11-7082 or the Caspase-1 inhibitor AC-YVAD-CMK along with E2 before undergoing the H/R procedure. Compared with the H/R + E2+ICI group, pretreatment with Bay11-7082 significantly preserved cell viability and reduced LDH release and TUNEL-positive cells (Figure 3). Furthermore, Bay11-7082 treatment resulted in a decrease in Caspase-1 activity, as well as a reduction in the release of TXNIP, IL-1β, and IL-18 (Figure 4). Consistently, the expression levels of TXNIP, NLRP3, ASC, cleaved-Caspase-1, GSDMD, and IL-1β were also decreased in the Bay11-7082-treated group (Figure 5). Similarly, pretreatment with AC-YVAD-CMK also led to a significant preservation of cell viability, reduction in LDH release, and decrease in the TUNEL-positive cells (Figure 3). AC-YVAD-CMK treatment resulted in a decrease in Caspase-1 activity, as well as a reduction in the release of TXNIP, IL-1β, and IL-18 (Figure 4). Additionally, the expression levels of TXNIP, NLRP3, ASC, cleaved-Caspase-1, GSDMD, and IL-1β were decreased in the AC-YVAD-CMK-treated group (Figure 5). These results further support the notion that inhibiting pyroptosis is involved in the cardioprotective effect of E2.

## 4. Discussion

Myocardial I/R injury represents a clinically paradoxical phenomenon wherein the restoration of blood flow exacerbates cellular damage. While estrogen replacement therapy is recognized for ameliorating such injury, its precise molecular mechanisms require further elucidation. Our investigation demonstrates that OVX followed by coronary ligation in mice markedly elevates key regulators of pyroptosis—including TXNIP, NLRP3 inflammasome components (ASC), activated Caspase-1, and the effector GSDMD—alongside the inflammatory cytokine IL-1β, observations consistent with inflammasome activation pathways documented in other systems. Pretreatment with 17β-estradiol (E2) significantly attenuated both histological injury and this pyroptotic protein cascade. Complementary in vitro H/R models corroborated E2's cardioprotection, mechanistically linked to ER-dependent signaling, where ER antagonism abolished E2's benefits. Crucially, E2-mediated suppression of pyroptosis partially accounted for reduced cellular injury, aligning with emerging paradigms of PCD in I/R pathology. Collectively, these data establish NLRP3 inflammasome-mediated pyroptosis inhibition via ER activation as a novel pathway for estrogen's cardioprotection in female subjects.

Women exhibit attenuated progression of coronary atherosclerosis and delayed onset of MI compared to age-matched men—a cardioprotective advantage that undergoes significant reversal following menopause due to ovarian function cessation and estrogen depletion [27]. A substantial evidence confirms that endogenous estrogen critically mediates this sexual dimorphism, conferring robust protection against coronary artery disease [28]. This protection extends to I/R injury contexts, with clinical and experimental studies consistently demonstrating heightened male vulnerability [29]. Mechanistic insights from Bae and Zhang further revealed superior functional recovery and reduced IS in female hearts using Langendorff perfusion models [29]. As the predominant bioactive estrogen, 17β-estradiol (E2) drives these effects: acute administration in OVX rabbits reduced IS [30], while Anderson et al. documented E2's normalization of ionic homeostasis and improved ventricular function in isolated hearts [31]. In line with these earlier investigations, the current study revealed that E2 replacement therapy led to a significant reduction in IS, TUNEL-positive cells, as well as levels of plasma LDH and CK-MB. Consistent with the in vivo findings, our study demonstrated that E2 pretreatment notably preserved cell viability, reduced LDH release, and decreased the number of TUNEL-positive cardiomyocytes in the H/R injury model.

The biological actions of E2, the predominant endogenous estrogen, are primarily mediated through its binding to specific nuclear receptors—estrogen receptor-α (ERα) and ERβ [32]. Both receptor subtypes exhibit comparable affinity for E2 [17] and have been independently implicated in mediating estrogen's cardioprotective effects against myocardial I/R injury [33, 34]. To directly investigate receptor involvement, we employed ICI 182780 (fulvestrant), a nonselective ER antagonist that downregulates receptor activity without agonist effects [30]. When cardiomyocytes were pretreated with this antagonist 24 h prior to H/R challenge, the protective effects of E2 were substantially attenuated. This pharmacological evidence conclusively demonstrates that estrogen's cardioprotection requires intact receptor signaling, establishing ER-dependent pathways as fundamental to mitigating I/R injury in cardiac tissue.

Apoptosis represents the most extensively documented form of PCD in myocardial I/R injury. Previous studies have established that E2 confers protection against I/R damage through antiapoptotic mechanisms [35]. Notably, emerging research indicates that pyroptosis—a distinct inflammatory cell death pathway—also contributes significantly to I/R pathology [36]. Critically, while estrogen's antiapoptotic effects are well documented, its potential regulation of pyroptosis via the TXNIP–NLRP3 axis remained unexplored prior to this investigation—representing a fundamental knowledge gap in understanding estrogenic cardioprotection. The TXNIP–NLRP3 interaction, a known initiator of pyroptotic signaling [12, 37], served as our mechanistic focus, employing established pyroptosis markers (TUNEL^+^ cells, cleaved Caspase-1) [38, 39]. We confirmed significant pyroptosis activation in I/R and H/R models, accompanied by upregulation of TXNIP/NLRP3, ASC, IL-1β, and GSDMD. Crucially, E2 replacement therapy not only attenuated I/R injury but selectively suppressed this TXNIP–NLRP3–pyroptosis cascade, evidenced by a coordinated downregulation of these mediators. This suppression proved estrogen receptor-dependent, as cotreatment with ICI 182780 partially reversed both cytoprotection and pathway inhibition. These findings establish TXNIP–NLRP3-mediated pyroptosis as a previously unrecognized mechanistic target of estrogen—distinct from its canonical antiapoptotic actions—thereby advancing our understanding of E2's cardioprotective repertoire.

Caspase-1 activation serves as the indispensable trigger for pyroptotic cell death [40]. This mechanistic understanding gains translational relevance from compelling evidence demonstrating that pharmacological inhibition of Caspase-1 during reperfusion salvages ischemic myocardium [41, 42]. To directly interrogate pyroptosis involvement in estrogen's protection, we employed the selective caspase-1 inhibitor AC-YVAD-CMK. Our experiments yielded three critical observations: First, E2 treatment potently suppressed Caspase-1 cleavage—an effect attenuated by coadministration of estrogen receptor antagonist ICI 182780, confirming the receptor dependence of this regulation. Second, AC-YVAD-CMK administration not only phenocopied E2's cardioprotective effects but fully restored its efficacy when estrogen receptor signaling was pharmacologically blocked. Third, this functional rescue extended to all pyroptotic markers examined, including DNA fragmentation, GSDMD processing, and cytokine release. Collectively, these data establish Caspase-1 as the essential executioner downstream of estrogen receptors in the TXNIP–NLRP3 pathway.

Building upon the established role of Caspase-1 in pyroptosis execution, we further interrogated NLRP3's upstream position in this pathway using BAY11-7082—a specific NLRP3 activation inhibitor with well-documented efficacy [39, 43]. In complementary in vitro experiments, E2 treatment profoundly suppressed NLRP3 inflammasome formation and subsequent pyroptotic cell death, significantly mitigating HR injury in cardiomyocytes. Crucially, this protective effect demonstrated estrogen receptor dependence, as coadministration of ICI 182780 partially reversed E2's inhibition of NLRP3 activation. When BAY11-7082 was introduced alongside E2 and ICI 182780, it effectively compensated for the compromised estrogen receptor signaling, restoring cardiomyocyte protection through a direct NLRP3 blockade. This pharmacological rescue manifested as a preserved cellular viability, attenuated LDH release, and coordinated downregulation of pyroptotic executors IL-1β and GSDMD. Collectively, these observations definitively position the TXNIP–NLRP3 axis as the mechanistic conduit through which estrogen exerts its antipyroptotic cardioprotection during ischemic injury.

## 5. Conclusions

**Figure 6:**
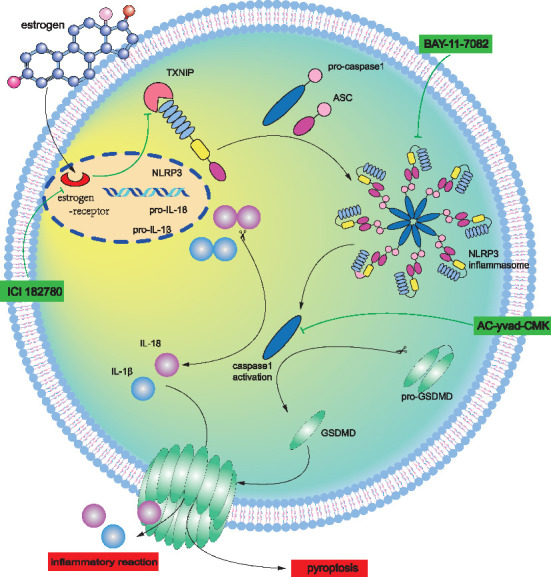
Estrogen alleviates myocardial I/R injury by inhibiting NLRP3 inflammasome-mediated pyroptosis. E2 alleviated myocardial I/R injury via its receptor ER to downregulate TXNIP–NLRP3 and further inhibit Caspase-1, thereby suppressing ASC, and IL-18, IL-1β, and GSDMD.

Our study provides compelling evidence that E2 plays a significant role in reducing myocardial I/R injury. We also elucidate the underlying mechanism by which E2 exerts its protective effects through its receptor ER, leading to the downregulation of TXNIP–NLRP3 signaling. This downregulation subsequently inhibits Caspase-1 activation, resulting in the suppression of ASC, IL-18, IL-1β, and GSDMD. Overall, our findings suggest that E2 alleviates myocardial I/R injury by effectively inhibiting NLRP3 inflammasome-mediated pyroptosis (Figure 6).

Our study has certain limitations that should be acknowledged. Firstly, the use of ER antagonist and pyroptosis inhibitors was limited to in vitro experiments only, which may not fully reflect the in *vivo* situation. Secondly, we did not investigate the underlying molecular mechanisms between ER and TXNIP due to laboratory constraints. Thirdly, the sample sizes and triplicate experimental repeats may limit extrapolation. Therefore, future studies utilizing ER or TXNIP gene knockout mice could provide further insights into the interaction between ER and TXNIP.

## Data Availability

The data that support the findings of this study are available from the corresponding authors upon reasonable request.

## Nomenclature

E2: 17β-estrogen
I/R: Ischemia/reperfusion
MI: Myocardial infarction
PCD: Programmed cell death
IL-1β: Interleukin-1β
IL-18: interleukin-18
GSDMD: Pro-Gasdermin D
NLRP3: Nucleotide-binding domain, leucine-rich-containing family, pyrin domain-containing-3
ASC: Apoptosis-associated speck-like protein
TXNIP: Thioredoxin-interacting protein
OVX: Ovariectomy
LDH: Lactate dehydrogenase
CK-MB: Creatine kinase-MB
H/R: Hypoxia/reoxygenation
LAD: Left coronary artery
TTC: Triphenyl tetrazolium chloride
AAR: Area at risk
IS: Infarct size
NVMs: Neonatal ventricular myocytes
EDTA: Ethylene diamine tetraacetic Acid
DMEM: Dulbecco's Modified Eagle's Media
FBS: Fetal bovine serum
